# Comparison of metagenomic next-generation sequencing using cell-free DNA and whole-cell DNA for the diagnoses of pulmonary infections

**DOI:** 10.3389/fcimb.2022.1042945

**Published:** 2022-11-10

**Authors:** Ping He, Jing Wang, Rui Ke, Wei Zhang, Pu Ning, Dexin Zhang, Xia Yang, Hongyang Shi, Ping Fang, Zongjuan Ming, Wei Li, Jie Zhang, Xilin Dong, Yun Liu, Jiemin Zhou, Han Xia, Shuanying Yang

**Affiliations:** ^1^ Department of Pulmonary and Critical Care Medicine, The Second Affiliated Hospital of Xi’an Jiaotong University, Xi’an, China; ^2^ Department of Scientific Affairs, Hugobiotech Co., Ltd., Beijing, China

**Keywords:** MNGs, cell-free DNA, whole-cell DNA, pulmonary infection, BALF

## Abstract

Although the fast-growing metagenomic next-generation sequencing (mNGS) has been used in diagnosing infectious diseases, low detection rate of mNGS in detecting pathogens with low loads limits its extensive application. In this study, 130 patients with suspected pulmonary infections were enrolled, from whom bronchoalveolar lavage fluid (BALF) samples were collected. The conventional tests and mNGS of cell-free DNA (cfDNA) and whole-cell DNA (wcDNA) using BALF were simultaneously performed. mNGS of cfDNA showed higher detection rate (91.5%) and total coincidence rate (73.8%) than mNGS of wcDNA (83.1% and 63.9%) and conventional methods (26.9% and 30.8%). A total of 70 microbes were detected by mNGS of cfDNA, and most of them (60) were also identified by mNGS of wcDNA. The 31.8% (21/66) of fungi, 38.6% (27/70) of viruses, and 26.7% (8/30) of intracellular microbes can be only detected by mNGS of cfDNA, much higher than those [19.7% (13/66), 14.3% (10/70), and 6.7% (2/30)] only detected by mNGS of wcDNA. After in-depth analysis on these microbes with low loads set by reads per million (RPM), we found that more RPM and fungi/viruses/intracellular microbes were detected by mNGS of cfDNA than by mNGS of wcDNA. Besides, the abilities of mNGS using both cfDNA and wcDNA to detect microbes with high loads were similar. We highlighted the advantage of mNGS using cfDNA in detecting fungi, viruses, and intracellular microbes with low loads, and suggested that mNGS of cfDNA could be considered as the first choice for diagnosing pulmonary infections.

## Introduction

Pulmonary infections are highly prevalent diseases with considerable morbidity and mortality in individuals of all ages (Griffin et al., 2013; Collaborators GBDCoD, 2017). As the third leading causes of years of life lost, about 2.4 million patients per year died of pulmonary infections globally (Collaborators GBDCoD, 2017). Various pathogens can cause such infections (Kradin and Digumarthy, 2017; Lin et al., 2021), with the presenting symptoms of fever, cough, sputum production, dyspnoea, pleuritic chest pain, and so on (Ruiz et al., 2000). However, due to the similar clinical manifestations among patients infected by different kinds of pathogens, the accurate and timely etiological diagnosis are always difficult for clinicians (Cunha, 2006; Sheu et al., 2010). Conventional tests for diagnoses of pulmonary infections, including time-consuming culture methods with low positive rate, polymerase chain reaction (PCR) based on prior hypothesis of the target, and serology tests with interpretational difficulties, are not satisfactory (Carroll and Adams, 2016; Buchan et al., 2021). Delay and misdiagnosis of pulmonary infections can lead to disease progression, resulting in worse prognoses and even death (Garnacho-Montero et al., 2018). More rapid and accurate methods for clinical diagnoses of pulmonary infections are needed.

Unbiased metagenomic next-generation sequencing (mNGS) has been increasingly applied in diagnosing multiple infectious diseases, such as meningitis and sepsis (Chiu and Miller, 2019; Gu et al., 2021), exhibiting significant advantages over conventional methods (Chen et al., 2021; Chen et al., 2021). mNGS of both cell-free DNA (cfDNA) and whole-cell DNA (wcDNA) are being used (Han et al., 2020). Compared to wcDNA extraction, cfDNA is extracted from extremely low-cellularity supernatant of samples (Szilagyi et al., 2020). Besides, cfDNA extraction is considered to decrease the load of pathogen DNA in the sample, especially for intracellular pathogens, but it can avoid DNA degradation caused by processes that wcDNA extraction requires. However, few investigations have been performed to evaluate diagnostic values of mNGS using cfDNA and wcDNA.

In this study, a total of 130 patients with suspected pulmonary infections were enrolled. The remaining bronchoalveolar lavage fluid (BALF) samples from those patients were used for mNGS of cfDNA and wcDNA. Our aim was to evaluate performance of mNGS using BALF in the clinical diagnosis of pulmonary infections against conventional methods, and effectiveness of mNGS using cfDNA and wcDNA was also compared.

## Methods

### Patient recruitment and study design

Patients with suspected pulmonary infections admitted to Department of Pulmonary and Critical Care Medicine of The Second Affiliated Hospital of Xi’an Jiaotong University from September 2019 to September 2021 were enrolled. The diagnosis of pulmonary infection was based on 1) new-onset radiological findings on chest X-ray or computed tomography (CT) and 2) at least one of the following typical clinical characteristics: a) new-onset cough, sputum production, dyspnoea, chest pain, or exacerbation of existing respiratory symptoms; b) fever; c) clinical signs of lung consolidation or moist rales; d) peripheral leukocytosis (>10×10^9^/L) or leucopenia (<4×10^9^/L).

Conventional diagnostic tests and mNGS were performed simultaneously. Conventional diagnostic tests used in this study included culture, antibody measurement, PCR, Xpert, and pulmonary histopathology. Physical information and clinical details were investigated. Remaining BALF sample from each enrolled patient was collected into a 5 mL sterile tube transported to Hugobiotech (Hugobiotech, Beijing, China) immediately for mNGS of cfDNA and wcDNA.

### Sampling and mNGS sequencing

According to manual of QIAamp DNA Micro Kit (QIAGEN, Hilden, Germany), cfDNA and wcDNA were extracted. For cfDNA extraction, BALF supernatant obtained by centrifugation was used for subsequent protocols, while BALF sample was directly used for wcDNA extraction (bead-beating method) without centrifugation. Before library construction (QIAseq Ultralow Input Library Kit, QIAGEN, Hilden, Germany), we tested concentration and quality of cfDNA and wcDNA using Qubit 4.0 (Thermo Fisher Scientific, MA, USA). Qualified libraries were sequenced on Nextseq 550 platform (Illumina, San Diego, USA). Negative controls using sterile deionized water and positive controls using synthesize fragments with known quantities were established for each batch of experiments using the same wet lab procedures and bioinformatics analysis as the clinical samples.

### Bioinformatics pipeline

Clean reads were obtained by removing adapter and low-quality and short reads (<35bp) from raw data generated by sequencing. Human sequences were excluded by mapping to the human reference genome (hg38) using bowtie2. The remaining clean reads were then blasted against a microbial Pan-genome database which was constructed based on the published microbial genome databases, including database of National Center for Biotechnology Information (ftp://ftp.ncbi.nlm.nih.gov/genomes/).

### Statistical analysis

Medians, interquartile ranges (IQRs), and 95% confidence interval (CI) were calculated using IBM SPSS 25.0. Sensitivity, specificity, positive predicative value (PPV), negative predicative value (NPV), and total coincidence rate (TCR) of mNGS were calculated against clinical diagnoses. Bar charts and heatmap were generated using R 4.1.1. Comparison with *P* value of < 0.05 was considered statistically significant. The number of microbes and base-2 logarithm of reads per million (RPM) detected by mNGS of cfDNA divided by that detected by mNGS of wcDNA for the same microbe were used to draw scatter plot, and the dots of >0 represented that the numbers of RPM detected by mNGS of cfDNA were higher than those detected by mNGS of wcDNA for the microbes. We set different thresholds for RPM (RPM of 25, 50, 100, 200, and 500) to divide the microbes into groups of high and low loads. The microbe with the number of RPM detected by mNGS of cfDNA or wcDNA higher than the threshold was divided into the high load group, while the others were divided into low load group.

## Results

### Patient characteristics

A total of 130 patients were enrolled in our study, including 81 males and 49 females. The age of these patients ranged from 20 to 67 years old, with a median age of 54 years old (**Table 1**). Most of patients (86.1%, 112/130) had underlying diseases, such as cardiovascular diseases (21), cerebrovascular diseases (13), hepatopathy (24), tumors (29), diabetes (18), autoimmunity disease (11), and anemia (13). According to mNGS results, conventional tests, and therapeutic effects, 121 and 9 patients were finally diagnosed as infectious and non-infectious diseases, respectively. The most common microbes detected were bacteria (85/121), followed by fungi (27/121) and viruses (15/121). Besides, co-infections were found in 21 patients (**Table 2**).

**Table 1:** Clinical characteristic of enrolled patients.

Clinical characteristics		Count (percent) / Median(IQR)[min,max](95% CI)
Gender	Male	81 (62.3%)
	Female	49 (37.7%)
Age		54 (20.75)[20,67](50.8~56.2)
Cough		112 (86.1%)
Expectoration		98 (75.4%)
Dyspnea		74 (56.9%)
Hydrothorax		17 (10%)
Intensive care unit		6 (4.6%)
Underlying diseases		112 (86.1%)
Autoimmunity disease		11 (8.5%)
Cardiovascular disease		21 (16.2%)
Cerebrovascular disease		13 (10%)
Diabetes		18 (13.8%)
Chronic obstructive pulmonary disease		8 (6.2%)
Pulmonary sarcoidosis		3 (2.3%)
Nephrosis		9 (6.9%)
Hepatopathy		24 (18.5%)
Asthma		7 (5.4%)
Tumors		29 (22.3%)
Anemia		13 (10%)

IQR, interquartile ranges; CI, confidence interval.

**Table 2:** The types of infections of enrolled patients based on clinical diagnosis.

Infections	Case number
Bacteria	66
Fungi	14
Virus	3
Bacteria + Fungi	8
Bacteria + Virus	6
Fungi + Virus	1
Bacteria + Fungi + Virus	4
Mycoplasma	3
Chlamydia	2
Bacteria + Mycoplasma	1
Mycoplasma + Chlamydia + Virus	1
Indefinite clinical diagnosis	12
Non-infected	9

### Potential pathogen profiles

**Figure 1:**
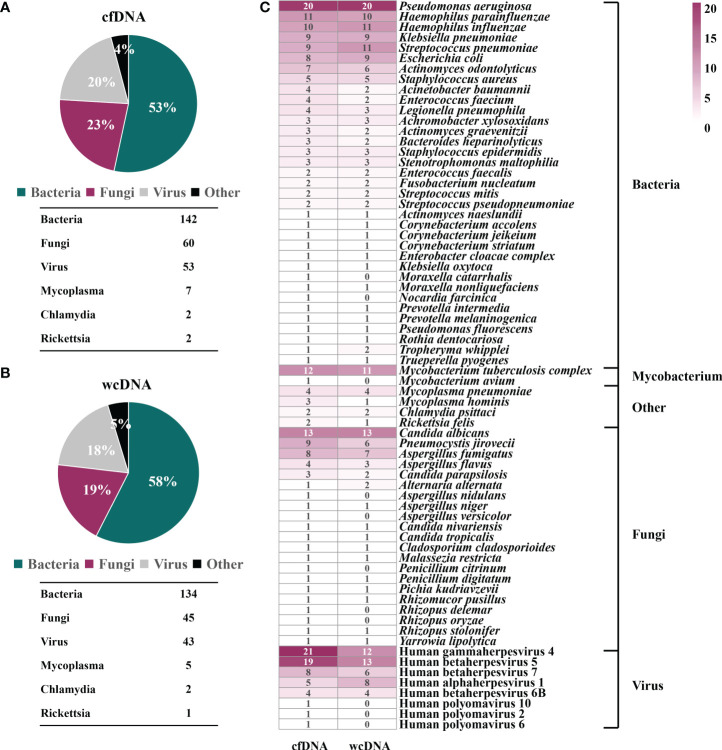
Potential pathogen profiles detected by mNGS of cfDNA and wcDNA. **(A)**, Microbial composition revealed by mNGS of cfDNA. **(B)**, Microbial composition revealed by mNGS of wcDNA. **(C)**, Comparison of mNGS using cfDNA and wcDNA in detecting microorganisms. The number in the box is the total number of patients from which some microorganism was detected.

mNGS of cfDNA identified bacteria (*n*=142), fungi (*n*=60), viruses (*n*=53), mycoplasmas (*n*=7), chlamydiae (*n*=2), and rickettsiae (*n*=2), most of which were also detected by mNGS of wcDNA (134 bacteria, 45 fungi, 43 viruses, 5 mycoplasmas, 2 chlamydiae, and 1 rickettsiae) (**Figures 1A, B**). Besides, the dominant microbes identified by mNGS of cfDNA and wcDNA were almost accordant. The main bacteria were *Pseudomonas aeruginosa*, *Haemophilus parainfluenzae*, *Haemophilus influenza*, *Klebsiella pneumoniae*, *Streptococcus pneumoniae*, and *Escherichia coli*, and fungi were *Candida albicans*, *Pneumocystis jirovecii*, and *Aspergillus fumigatus*. A total of 8 viruses were detected, with the detection of Human betaherpesvirus 5 and Human gammaherpesvirus 4 at the highest frequencies (**Figure 1C**).

However, some bacteria (2: *Moraxella catarrhalis* and *Nocardia farcinica*), fungi (5: *Aspergillus nidulans*, *Aspergillus versicolor*, *Penicillium citrinum*, *Rhizopus delemar*, and *Rhizopus oryzae*), and viruses (3: Human polyomavirus 10, Human polyomavirus 2, and Human polyomavirus 6), and *Mycobacterium avium* were only detected by mNGS of cfDNA. Although there were no significant difference in overall detection of microorganisms, more microbes identified by mNGS of cfDNA can provide more effective reference for clinicians.

### mNGS performance

**Figure 2:**
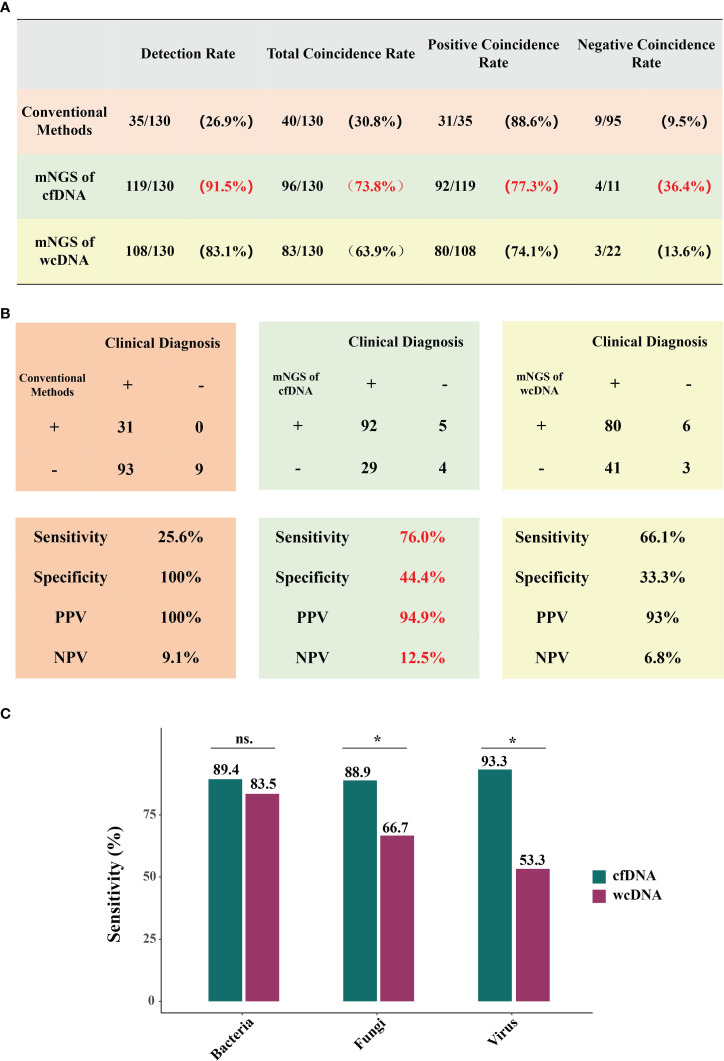
Performance of mNGS using cfDNA/wcDNA and conventional methods. **(A, B)** show the coincidence rates, sensitivity, specificity, PPV, and NPV of conventional methods, mNGS of cfDNA, and mNGS of wcDNA. PPV and NPV represent positive predictive value and negative predictive value, respectively. **(C)**, Comparison of mNGS using cfDNA and wcDNA at bacterial, fungal, and viral levels. *P < 0.05; ns: no significance.

From case perspective, the performance of mNGS using cfDNA was better than that of mNGS using wcDNA. Conventional methods only detected microorganisms from 26.9% of BALF samples (35/130). Conversely, detection rate of mNGS using cfDNA reached 91.5% (119/130), higher than that of mNGS using wcDNA (108/130, 83.1%). Besides, sensitivity (76.9%), specificity (44.4%), PPV (94.9%), and NPV (12.5%) of mNGS using cfDNA were all higher than those (66.1%, 33.3%, 93%, and 6.8%) of mNGS using wcDNA (**Figure 2B**). Most importantly, TCR of mNGS using cfDNA against final clinical diagnoses was 73.8%, higher than those of mNGS using wcDNA (63.9%) and conventional methods (30.8%) (**Figure 2A**).

Given mNGS advantages and microbial complexity, we further evaluated the efficiencies of mNGS in detecting bacterial, fungal, and viral infections. Among the 85 patients with bacterial infection, mNGS of cfDNA (89.4%) and wcDNA (83.5%) respectively detected microbes from 76 and 71 patients (**Figure 2C**). However, mNGS of cfDNA exhibited better performance in detecting both fungi (88.9%, 24/27) and viruses (93.3%,14/15) than mNGS of wcDNA (66.7% and 53.3%, respectively) (**Figure 2C**). These results further indicate that mNGS of cfDNA is much more suitable for diagnosing pulmonary infections than mNGS of wcDNA, driving us to dig out in-depth reasons from perspectives of total microorganisms and RPM detected.

### Differences in numbers of RPM detected

**Figure 3:**
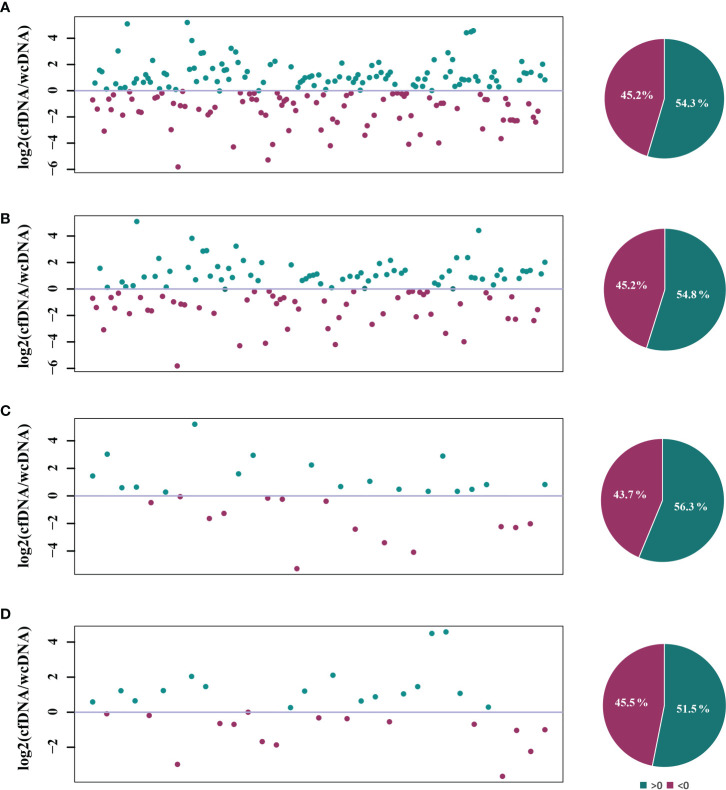
Comparison of the RPM detected by mNGS of both cfDNA and wcDNA. **(A)**, All microbes detected by mNGS. **(B)**, Bacteria detected by mNGS. **(C)**, Fungi detected by mNGS. **(D)**, Viruses detected by mNGS. The number of microbes and base-2 logarithm of reads per million (RPM) detected by mNGS of cfDNA divided by that detected by mNGS of wcDNA for the same microbes were used to draw scatter plot, and the dots of >0 represented that the numbers of RPM detected by mNGS of cfDNA was higher than that detected by mNGS of wcDNA for the microbes. The microbes only detected by mNGS of cfDNA or wcDNA were summarized in **Table 3**.

To evaluate the detection efficiencies of mNGS, comparison of difference in detected RPM between mNGS of cfDNA and wcDNA was further performed by infection types. There were 206 microbes detected by mNGS of both cfDNA and wcDNA. For most of microbes (54.3%), mNGS of cfDNA detected more RPM than mNGS of wcDNA (**Figure 3A**). Similar trends were also found in detecting bacteria (54.8%), fungi (56.3%), and viruses (51.5%) (**Figures 3B-D**). These results show that mNGS of cfDNA can capture more reads from most of microbes than mNGS of wcDNA.

Differences in clinical diagnostic value between the two methods are caused by the heterogeneity of microbes detected, rather than the uniformity. Among the 308 microbes, 69 (22.4%) and 33 (10.7%) microbes were only detected by mNGS of cfDNA and wcDNA, respectively (**Table 3**). The 31.8% (21/66) of fungi, 38.6% (27/70) of viruses, and 26.7% (8/30) of intracellular microbes (viruses were not included) were only detected by mNGS of cfDNA, much higher than those (19.7% (13/66), 14.3% (10/70), and 6.7% (2/30), respectively) by mNGS of wcDNA (**Table 3**). Besides, the number of RPM only detected by mNGS of cfDNA ranged from 3 to 12010, lower than that (from 2 to 30446) by mNGS of wcDNA. These results indicate that the better performance of mNGS using cfDNA might be caused by the successful detection of microbes with low loads, especially for fungi, viruses, and intracellular microbes.

**Table 3:** Statistics of microbes only detected by mNGS of cfDNA or wcDNA.

	Only detected	All Microbes	Bacteria	Fungi	Viruses	Intracellular Microbes
**Count**	**by mNGS of cfDNA**	69/308	18/152	21/66	27/70	Aug-30
**(Percentage)**		-22.40%	-11.80%	-31.80%	-38.60%	-26.70%
**RPM**		(3~12010)	(6~12010)	(3~92)	(4~67)	(4~1875)
**Count**	**by mNGS of wcDNA**	33/308	10/152	13/66	Oct-70	Feb-30
**(Percentage)**		-10.70%	-6.60%	-19.70%	-14.30%	-6.70%
**RPM**		(2~30446)	(6~30446)	(7~851)	(2~843)	(6, 17)

### Identifying microbes with low loads

**Figure 4:**
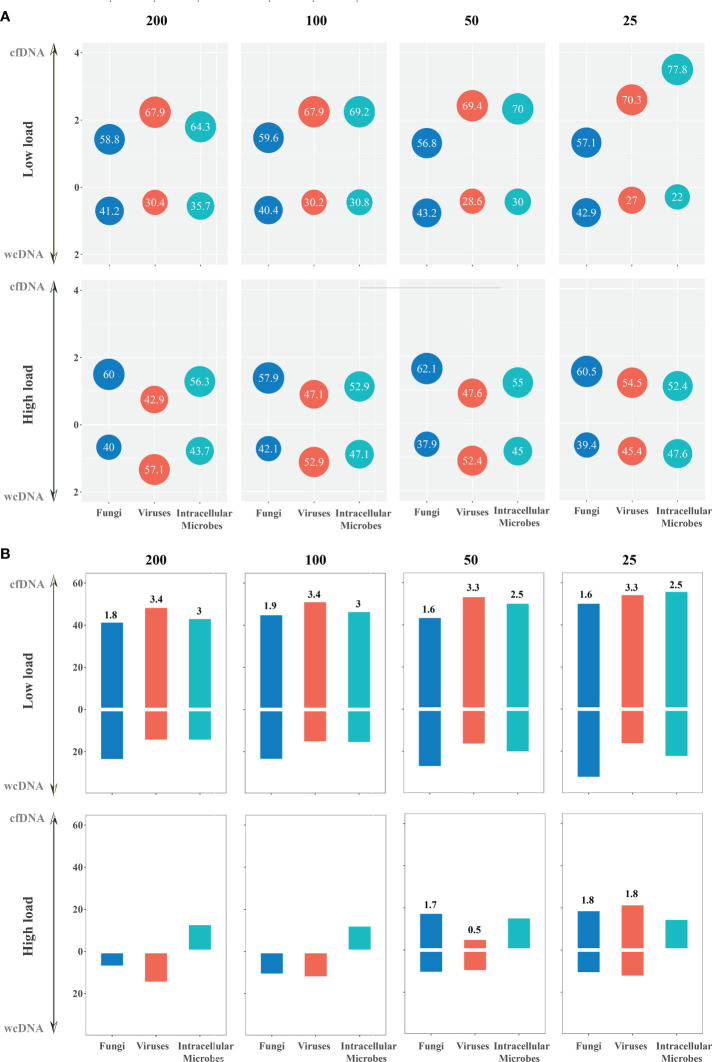
The detection of mNGS using cfDNA and wcDNA at different thresholds (RPM of 200, 100, 50, and 25). **(A)** Detection summary of fungi, viruses, and intracellular microbes by mNGS of both cfDNA and wcDNA at high and low loads. The number in the bubble above the horizontal axis is the ratio of the microbes with more RPM by mNGS of cfDNA than mNGS of wcDNA to the whole microbes at this threshold, while the number in the bubble below the horizontal axis is the ratio of the microbes with more RPM by mNGS of wcDNA than mNGS of cfDNA. Besides, vertical axis coordinate is the ratio of the numbers in the bubble. **(B)** Detection summary of fungi, viruses, and intracellular microbes only detected by mNGS of cfDNA or wcDNA at high and low loads. The column is the ratio of the microbes only detected by mNGS of cfDNA or wcDNA to the whole microbes at this threshold. The number above the column is the ratio of the microbes only detected by mNGS of cfDNA divided by that only detected by mNGS of wcDNA.

Based on the assumption that sequencing process did not influence the detection of mNGS using both cfDNA and wcDNA, we proposed that the level of microbial loads can be directly reflected by the numbers of RPM. Accordingly, we set different thresholds for RPM to evaluate the ability of mNGS using cfDNA and wcDNA to detect microbes. For bacteria with high loads at different thresholds (RPM of 100, 200, and 500), mNGS of cfDNA detected more RPM than mNGS of wcDNA in 58.0%-63.7% of bacteria, while the two methods had the similar abilities to detect bacteria with low loads (**Figure 4** and **Table S1**).

Better performance of mNGS using cfDNA was also observed in detecting fungi and viruses with low loads. For fungi with low loads at different thresholds (RPM of 25, 50, 100, and 200), the numbers of RPM from about 60% of fungi detected by mNGS of cfDNA were higher than those detected by mNGS of wcDNA. Most importantly, the number of fungi only detected by mNGS of cfDNA was about one time higher than that by mNGS of wcDNA. However, no significant difference in the number of RPM between mNGS of cfDNA and wcDNA was found in detecting fungi with high loads (**Figure 4** and **Table S1**). A similar trend was found in detecting viruses. These results unravel that the better performance of mNGS using cfDNA is definitely caused by the successful detection of fungi and viruses with low loads.

### Identifying intracellular microbes

Given the long-held conflict in location of proliferation and infection between intracellular and extracellular pathogens, we summarized the performance of mNGS in detecting intracellular microbes (viruses were not included). From 27 patients, *Mycobacterium tuberculosis* complex, *Mycoplasma hominis*, *Legionella pneumophila*, *Chlamydia psittaci*, and *Rickettsia felis* were detected with total number of 30. The detection rate of mNGS using cfDNA was 92.6% (25/27), slightly higher than that of mNGS using wcDNA (81.5%, 22/27). About 26.7% and 6.7% of intracellular microbes were only detected by mNGS of cfDNA and wcDNA, respectively (**Table 3**).

For intracellular microbes with both high and low loads at different thresholds (RPM of 25, 50, 100, 200, and 500), the numbers of RPM from 50%-77.8% of mircobes detected by mNGS of cfDNA were higher than those detected by mNGS of wcDNA (**Figure 4** and **Table S1**). Besides, the number of microbes with low loads only detected by mNGS of cfDNA was more than two times higher than that only detected by mNGS of wcDNA. Interestingly, all of intracellular microbes with high loads at different thresholds detected by mNGS of wcDNA were detected by mNGS of cfDNA, but some intracellular microbes (species number of 1-3) detected by mNGS of cfDNA cannot be detected by mNGS of wcDNA at all. The above results provided sufficient evidences for the better performance of mNGS using cfDNA in detecting intracellular microbes, especially for those with low loads.

## Discussion

This is the first report on evaluating performance of mNGS using cfDNA and wcDNA of BALF samples in diagnosing pulmonary infections. The highest detection rate (91.5%) and TCR (73.8%) were found by mNGS of cfDNA, followed by mNGS of wcDNA (83.1% and 63.9%) and conventional methods (26.9% and 30.8%). We provided sufficient evidences for that the better performance of mNGS using cfDNA than that of mNGS using wcDNA is definitely caused by the successful detection of microbes with low loads, especially for fungi, viruses, and intracellular microbes.

To evaluate the performance of mNGS in diagnosing pulmonary infections, mNGS tests were performed using cfDNA and wcDNA without host depletion. Host depletion methods, such as differential lysis method, can filter human DNA (Ji et al., 2020), increasing pathogen DNA ratio (Thoendel et al., 2018; Ji et al., 2020; Gu et al., 2021) at the expense of some viruses, parasites, and bacteria (Ji et al., 2020). Besides, host depletion methods lose the cfDNA in supernatant, bring in contamination of engineered strains from reagents (Gu et al., 2021), and decrease the detection rates of pathogens (Han et al., 2021). The long-held conflict in location of proliferation and infection between intracellular and extracellular pathogens (Casadevall and Fang, 2020) challenged whether mNGS of cfDNA can accurately detect causative pathogens. Accordingly, mNGS tests of wcDNA without host depletion used in this study can be considered as a suitable contrast to the mNGS tests of cfDNA, and the use of the both tests ensures reliability and accuracy of our data.

mNGS of cfDNA exhibited better performance in diagnosing pulmonary infections than mNGS of wcDNA and conventional methods. Low detection efficiencies and accuracies of conventional methods, such as culture (Rong et al., 2020) and antibody test (Shibata et al., 2020), hindered accurate and comprehensive detection of pathogens. Conversely, mNGS of cfDNA has been proven to be a promising tool for detecting pathogens in body fluids with high sensitivities (75-91%) and specificities (81-100%) (11). In addition, the sensitivity of mNGS is determined by pathogen DNA ratio in sample. However, wcDNA extraction from BALF sample by cell fragmentation without host depletion must increase the release of human DNA (11). CfDNA was directly extracted from low-cellularity supernatant of BALF samples (14), resulting in that pathogen DNA ratio of cfDNA might be higher than that of wcDNA for the same BALF sample, and the sensitivity of mNGS using BALF cfDNA reached up to about 90% (14). The above may be the reasons why the performance of mNGS using cfDNA is better than that of mNGS using wcDNA and conventional methods.

Obvious advantage of mNGS using cfDNA over mNGS using wcDNA is reflected in detecting pathogens with low loads, rather than high loads. We propose that the better performance of mNGS using cfDNA is attributed to its advantage in DNA extraction and bioinformatics analysis for trace pathogens. Although sample pretreatment for host depletion is not included in this study, wcDNA extraction involves cell wall lysis (Wilson et al., 2019), increasing the risk of DNA degradation (Thoendel et al., 2018; Gu et al., 2021) and decreasing DNA recovery rate of pathogens with low loads (Han et al., 2021), rather than high loads. Mild extraction of cfDNA without cell wall lysis should have few effect on the DNA recovery, which has been confirmed by successful detection of trace pathogens from cerebrospinal fluid using mNGS of cfDNA (Ji et al., 2020).

Furthermore, high human DNA ratio can reduce the denoising performance of bioinformatics algorithm in dry lab pipeline of mNGS and influence subsequent pathogen identification (Miller et al., 2019), especially for trace pathogens (Ji et al., 2020), which was confirmed by low detection rate (~50%) (Zhang et al., 2020) and coincidence rate (~50%) (Wilson et al., 2019) of mNGS using CSF wcDNA without host depletion and high sensitivity (>90%) of mNGS using cfDNA (Gu et al., 2021). Besides, accuracy of bioinformatics algorithm used in this study has been demonstrated by successful identification of 2 reads from desired pathogen using mNGS of wcDNA with host depletion (Wu et al., 2020). Equipped with the same bioinformatics algorithm, trace *M. tuberculosis* (RPM: 2.28) (Ji et al., 2020) was identified by mNGS of cfDNA, rather than mNGS of wcDNA. Lower DNA recovery rate and worse denoising performance of mNGS using wcDNA without host depletion comprehensively reduce its accuracy in detecting trace pathogens.

mNGS of cfDNA can be fully competent for detecting fungal and intracellular pathogens, challenging the opinion that process of cell wall lysis, such as bead-beating process, is necessary for DNA extraction (wcDNA) to ensure mNGS detection for those pathogens (Casadevall and Fang, 2020; Han et al., 2021). Adding bead-beating process was reported to significantly improve the detection of *Aspergillus fumigatus* by mNGS of wcDNA (Han et al., 2021). However, with the host immune attack (Arciola et al., 2018) and microbial autolysis (Wolf et al., 2017), DNA of intracellular and fungal pathogens can be released into body fluids in the form of cfDNA (Casadevall and Fang, 2020). Besides, we previously detected fungal (including *Aspergillus*) and intracellular pathogens consistent with clinical diagnosis from BALF samples using mNGS of cfDNA (Chen et al., 2021). Accordingly, extensive application of mNGS using cfDNA in diagnosing pulmonary infections could be expected.

## Limitations

Firstly, multi-center study should be performed to provide more effective data and avoid intrinsic bias. Secondly, mNGS of wcDNA with host depletion should be included to capture more difference caused by different sample processing methods. Thirdly, efficiency of mNGS using cfDNA in detecting mycobacteria and intracellular microbes can be further investigated on large scale. Furthermore, more detailed clinical information, such as therapeutic regimens before and after mNGS, can be collected to evaluate implications of mNGS for clinical reference.

## Conclusions

mNGS exhibited higher sensitivities and coincidence rates against clinical diagnosis than conventional methods in detecting microbes from patients with pulmonary infections. We emphasized the advantage of mNGS using BALF cfDNA with sufficient evidences in detecting microbes with low loads, especially for fungi, viruses, and intracellular microbes, and its extensive application in diagnosing pulmonary infections could be excepted.

## Data availability statement

The datasets presented in this study can be found in online repositories. The names of the repository/repositories and accession number(s) can be found below: https://ngdc.cncb.ac.cn/?lang=zh, PRJCA008662.

## Ethics statement

The studies involving human participants were reviewed and approved by the Clinical Research Ethics Committee of the second affiliated hospital of Xi’an Jiaotong University. The patients/participants provided their written informed consent to participate in this study.

## Author contributions

SY and HX designed the paper. PH and JW drafted the manuscript. RK, WZ, PN, DZ, XY, HS, PF, ZM, WL, JZ, XD and YL carried out the clinical care and management of the patients and performed the mNGS tests. JMZ analyzed the data. SY and HX revised the manuscript. All authors contributed to the article and approved the submitted version.

## Funding

This study was supported by Shaanxi Qinchuangyuan “Scientists and Engineers” Team Construction Project (No. 2022KXJ-82).

## Conflict of interest

JW, JMZ and HX were employed by Hugobiotech.

The remaining authors declare that the research was conducted in the absence of any commercial or financial relationships that could be constructed as a potential conflict of interest.

## Publisher’s note

All claims expressed in this article are solely those of the authors and do not necessarily represent those of their affiliated organizations, or those of the publisher, the editors and the reviewers. Any product that may be evaluated in this article, or claim that may be made by its manufacturer, is not guaranteed or endorsed by the publisher.
