# Supplementary material for: Comparison of metagenomic next-generation sequencing using cell-free DNA and whole-cell DNA for the diagnoses of pulmonary infections
Source: Front Cell Infect Microbiol. 2022 Nov 10;12:1042945. doi: 10.3389/fcimb.2022.1042945 (PMC9684712; doi:10.3389/fcimb.2022.1042945)
Supplement: Supplementary file 1 [file DataSheet_1.pdf]

Comparison of metagenomic next-generation sequencing using cell-free DNA and whole-cell DNA for the diagnoses of pulmonary infections

Ping He[1, a], Jing Wang[2, a], Rui Ke[1], Wei Zhang[1], Pu Ning[1], Dexin Zhang[1], Xia Yang[1], Hongyang Shi[1], Ping Fang[1], Zongjuan Ming[1], Wei Li[1], Jie Zhang[1], Xilin Dong[1], Yun Liu[1], Jiemin Zhou[2], Han Xia[2,*] Shuanying Yang[1,*]

[1]Department of Pulmonary and Critical Care Medicine, The Second Affiliated Hospital of Xi'an Jiaotong University, 157th Xiwu Road, Xi'an 710000, People's Republic of China.

[2]Hugobiotech Co., Ltd., Disheng East Road, Daxing District, Beijing 100176, People's Republic of China.

[a]P.H. and J.W. contributed equally.

*Correspondence: H.X. (xiahan@hugobioitech.com) and S.Y. (yangshuanying@xjtu.edu.cn).

TABLE S1 The detection of mNGS using cfDNA and wcDNA at different thresholds

Thresholds for RPM		cfDNA *vs.* wcDNA	Bacteria	Bacteria (only detected by cfDNA or wcDNA)	Fungi	Fungi (only detected by cfDNA or wcDNA)	Viruses	Viruses (only detected by cfDNA or wcDNA)	Intracellular Microbes	Intracellular Microbes (only detected by cfDNA or wcDNA)
500	**low load**	>	**32(47.8%)**	**15(22.4%)**					**11(68.8%)**	**7(43.8%)**
		<	**35(52.2%)**	**9(13.4%)**					**5(31.2%)**	**2(12.5%)**
	high load	>	54(63.5%)	3(3.5%)					7(50%)	1(7.1%)
		<	31(36.5%)	1(1.2%)					7(50%)	0
200	**low load**	>	**21(42%)**	**12(24%)**	**30(58.8%)**	**21(41.2%)**	**38(67.9%)**	**27(48.2%)**	**9(64.3%)**	**6(42.9%)**
		<	**29(58%)**	**8(16%)**	**21(41.2%)**	**12(23.5%)**	**17(30.4%)**	**8(14.3%)**	**5(35.7%)**	**2(14.3%)**
	high load	>	65(63.7%)	6(5.9%)	9(60%)	0	6(42.9%)	0	9(56.3%)	2(12.5%)
		<	37(36.3%)	2(2%)	6(40%)	1(6.7%)	8(57.1)	2(14.3%)	7(43.7%)	0
100	**low load**	>	**17(51.5%)**	**10(30.3%)**	**28(59.6%)**	**21(44.7%)**	**36(67.9%)**	**27(50.9%)**	**9(69.2%)**	**6(46.2%)**
		<	**16(48.5%)**	**8(24.2%)**	**19(40.4%)**	**11(23.4%)**	**16(30.2%)**	**8(15.1%)**	**4(30.8%)**	**2(15.4%)**
	high load	>	69(58%)	8(6.7%)	11(57.9%)	0	8(47.1%)	0	9(52.9%)	2(11.8%)
		<	50(42%)	2(1.7%)	8(42.1%)	2(10.5%)	9(52.9%)	2(11.8%)	8(47.1%)	0
50	**low load**	>			**21(56.8%)**	**16(43.2%)**	**34(69.4%)**	**26(53.1%)**	**7(70%)**	**5(50%)**
		<			**16(43.2%)**	**10(27%)**	**14(28.6%)**	**8(16.3)**	**3(30%)**	**2(20%)**
	high load	>			18(62.1%)	5(17.2%)	10(47.6%)	1(4.8%)	11(55%)	3(15%)
		<			11(37.9%)	3(10.3%)	11(52.4%)	2(9.5%)	9(45%)	0
25	**low load**	>			**16(57.1%)**	**14(50%)**	**26(70.3%)**	**20(54.1%)**	**7(77.8%)**	**5(55.6%)**
		<			**12(42.9%)**	**9(32.1%)**	**10(27%)**	**6(16.2%)**	**2(22.2%)**	**2(22.2%)**
	high load	>			23(60.5%)	7(18.4%)	18(54.5%)	7(21.2%)	11(52.4%)	3(14.3%)
		<			15(39.4%)	4(10.5%)	15(45.4%)	4(12.1%)	10(47.6%)	0
